# The hairy sole sign

**DOI:** 10.1016/j.jdcr.2023.09.004

**Published:** 2023-09-24

**Authors:** Jennifer J. Parker, Shayan Waseh, Sylvia Hsu

**Affiliations:** Department of Dermatology, Temple University Lewis Katz School of Medicine, Philadelphia, Pennsylvania

**Keywords:** hair shaft, migratory eruption

## Introduction

Cutaneous pili migrans (CPM) is an extremely rare skin condition that is characterized by a creeping eruption mimicking cutaneous larva migrans. In CPM, a hair shaft is embedded in the epidermis or reticular dermis, and it migrates with the patient’s movement. Removal of the hair shaft resolves the condition completely. A PubMed search for CPM revealed only 16 reports. We report a case in which CPM began after the patient ran a marathon.

## Case reports

**Fig 1:**
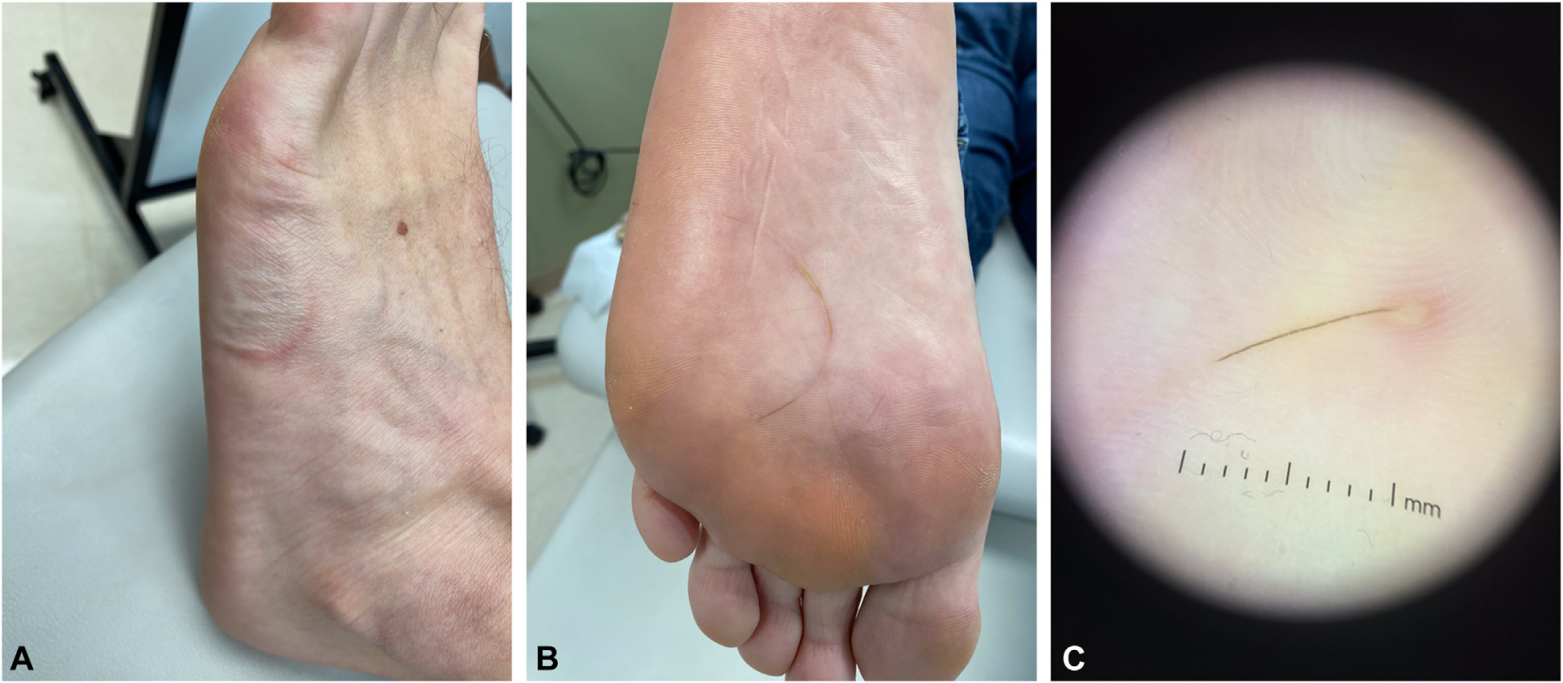
The image shows the left laterodorsal foot (**A**) and left sole (**B**) with a curvilinear streak and the dermatoscopic image of the foreign body (**C**).

A 50-year-old man presented with a 2-week history of a painful, annular eruption on his left foot. The eruption developed 2 days after running a marathon. The patient was treated with albendazole 400 mg daily for 3 days for presumed cutaneous larva migrans (CLM) by his local dermatologist. He noted minimal improvement with albendazole. On physical examination, there was a curvilinear streak on his left foot (Fig 1, *A* and *B*). Dermatoscopy revealed a linear object, most consistent with a hair shaft (Fig 1, *C*). Extraction of the hair resulted in immediate resolution of his foot pain. Given these findings, the patient was diagnosed with CPM.

## Discussion

CPM is an extremely rare condition that mimics CLM. However, instead of a hookworm that migrates in the skin as in CLM, a hair is implanted into the skin from trauma, and the hair migrates in the skin with the patient’s movement. Furthermore, CPM usually moves in a linear fashion in a single direction, whereas CLM moves in any direction and creates a serpiginous pattern.^1^ CPM has been reported to occur on the ankle, foot, breast, abdomen, face, and neck.^1^ In addition, CLM is usually itchy, whereas CPM is either asymptomatic or painful.^2^ CPM can be diagnosed by physical examination and completely treated by removal of the hair.

## Conflicts of interest

None disclosed.
